# D-optimal mixture design optimized solid formulation containing fruits extracts of *Momordica charantia* and *Abelmoschus esculentus*

**DOI:** 10.1371/journal.pone.0270547

**Published:** 2022-06-24

**Authors:** Emanuel L. Peter, Crispin D. Sesaazi

**Affiliations:** 1 Department of Innovation, Technology Transfer and Commercialization, National Institute for Medical Research, Dar Es Salaam, Tanzania; 2 Department of Pharmaceutical Sciences, Faculty of Medicine, Mbarara University of Science and Technology, Mbarara, Uganda; Tribhuvan University, NEPAL

## Abstract

Fruit extracts of *Momordica charantia* L. (Cucurbitaceae) and *Abelmoschus esculentus* (L.) Moench (Malvaceae) have shown promising antidiabetic activities in clinical trials. However, they remain underutilized due to insufficient standardization and lack of formulation containing their mixture. This study’s overall purpose was to develop and optimize a capsule dosage form containing dried fruit extracts *of M*. *charantia* and *A*. *esculentus*. The design of the experiment involved two steps; first, response surface methodology (RSM) with a five-level two-factor central composite rotatable design (CCRD) was employed to determine the optimal dose of a mixture of extracts for adequate glycemic control. The extract of *M*. *charantia* and *A*. *esculentus* were the independent variables while fasting plasma glucose (FPG) was the dependent factor. In the second step, a D-optimal mixture design was applied to study the interaction effect of the optimal dose and selected excipients on granules flowability and capsules’ disintegration time. Moreover, a second-order quadratic model determined the interrelationship of excipients and the desired capsules’ quality attributes. The validity of the predicted models was confirmed. The findings indicated that a combined dose of 175 *A*. *esculentus* and 281 *M*. *charantia* (mg/kg) significantly reduced the FPG level compared to vehicle at day 14 (mean difference -2.7 ± 0.21, *p* < 0.001). This dose was used to make a 600 mg capsule (DM083) with 76% drug loading. The DM083 had 40.4 ± 0.62 mg GAE/gDW total polyphenols, 12 peaks HPLC fingerprint, and 26.6 ± 4.75 min average disintegration time. Together, these findings showed that a mixture of *M*. *charantia* and *A*. *esculentus* fruit extracts could be formulated in a stable capsule dosage form with acceptable quality standards. Further biological studies such as toxicity assays and long-term efficacy studies of the developed capsules could be carried out before large-scale commercial production.

## Introduction

Herbal medicines use among individuals with diabetes mellitus (DM) has gradually increased [1]. This growing use of herbal medicine is attributed to several reasons, including; inadequate availability of conventional medicines, especially in the rural area, increased reports of failure and side effects associated with conventional antidiabetic agents; perceived herbal medicine affordability, widespread availability, and perceived safety and efficacy of herbal medicine [2–7]. Consequently, the public demand for convenient and quality herbal formulations has increased [8]. Furthermore, the forecasted exponential increase in the global prevalence of DM from the current 537 million adults (20–79 years) to 643 million by 2030 is likely to augment this demand [9]. Therefore, there is a need to develop suitable herbal dosage forms for DM.

*Momordica charantia* L. (Cucurbitaceae) is a popular vegetable in African countries, India, and China [10–12]. Pre-clinical studies showed that *M*. *charantia* preparations lower elevated blood glucose levels [13]. This action occurs through several mechanisms, including β-cell regeneration [14–16], insulin secretagogue [17, 18], enhancing peripheral glucose utilization, inhibiting glucose-6-phosphatase and fructose biphosphatase glucogenic enzymes [19], increasing adiponectin release [20], and inhibit 11β-Hydroxysteroid dehydrogenase type 1 among other [21, 22]. However, despite its versatility in managing DM as demonstrated in pre-clinical studies, earlier meta-analyses of clinical trials of *M*. *charantia* preparations identified no significant benefit to patients with DM [23, 24], with the most recent trials indicating low-quality evidence, citing inadequately standardized dosage forms as a foremost concern [25]. Moreover, studies found that combining plant species’ extracts better manages chronic conditions such as DM than a single extract [26]. Thus, the marginal antidiabetic effect of *M*. *charantia* preparations could be enhanced through mixing with appropriate plant species with known antidiabetic properties such as *Abelmoschus esculentus* (Malvaceae).

The extract, fractions, and pure compounds of *A*. *esculentus* inhibit α-glucosidase and α-amylase [27–29], potentiate pancreatic islets of Langerhans function [30], remove reactive oxygen species (ROS) in the pancreas via increased antioxidant enzymes (glutathione peroxidase (GPx), superoxide dismutase (SOD), glutathione (GSH), and catalase) [31–34].

Although the two plant species are widely available in the local communities and traditionally used as vegetables and herbal medicines to manage DM [35, 36], herbal formulations containing their combination have not been reported.

Herbal formulation development is complex because it involves incorporating large quantities of herbal extracts as active pharmaceutical ingredients and inert excipients. Thus, it requires a rigorous approach to select excipients that could improve the extract’s unfavorable physicochemical properties such as hygroscopicity and stickiness to meet the predetermined product’s critical quality attributes (CQAs). In recent years, response surface methodology (RSM) utilizing D-optimal mixture design has emerged as the preferred statistical formulation approach for analyzing the interrelations between mixture composition and CQAs [37–39]. The popularity of this statistical approach could be due to its reliability in reducing the time and cost of identifying desirable formulation compositions that meet the product’s quality criteria [40].

Oral solid dosage form such as capsules remains a popular means of administering medicine. This popularity could be due to their easy-to-use and manufacture, require fewer excipients than other solid dosage forms, they are tasteless hence can effectively mask the unpleasant taste or odor [41]. Therefore, this study used response surface methodology with a D-optimal mixture design to develop and optimize a capsule herbal dosage form containing a binary mixture of *M*. *charantia* and *A*. *esculentus* fruit extracts.

## Materials and methods

### Materials

Fresh fruits of *M*. *charantia* and *A*. *esculentus* were obtained from a vegetable garden along the northern bypass road at 0°34’53.0"S, 30°39’30.0"E, (Mbarara, Uganda), ethanol absolute, Molecular weight (Mwt) = 46.07 (VWR, chemicals, France), Sodium citrate 99% assay, Mwt = 294.10 (UNILAB Kenya Limited, Nairobi, Kenya), industrial-grade Sodium bicarbonate, Mwt = 84.01 (HiMedia Laboratories, Mumbai, India), Magnesium carbonate, Mwt = 84.31 (Thomas Baker Chemicals Limited, Mumbai, India), Lactose, Mwt = 342.30 (BDH laboratory suppliers, Kampala, Uganda), Talc (IDA, Merch, Eurolab, Belgium), Folin-Ciocalteu reagent (Labo Chemie Pvt Limited, Mumbai, India), HPLC grade gallic acid, Mwt = 170.12 (Labo Chemie Pvt Limited, Mumbai, India), Quercetin, Mwt = 302.24 (Sigma-Aldrich, St. Louis, USA). Also, HPLC grade; methanol, Mwt = 32.04, acetonitrile, Mwt = 41.05, and trifluoroacetic acid, Mwt = 114.02 were obtained from (Labo Chemie Pvt Limited, Mumbai, India), distilled water was in-house prepared. Other materials were used, including a shaking water bath (OLS 200, Grants, UK) and a rotary evaporator (RV 10D S99, IKA, German).

### Plant extract preparation

The *M*. *charantia* and *A*. *esculentus* plant specimens were processed and sent to the Department of Botany at Makerere University. Mr. Protase Rwaburindori, a plant taxonomist at Makerere university, identified and authenticated the plant specimens. Then, voucher specimens for *Abelmoschus esculentus* L. (Moench) (Malvaceae) and *Momordica charantia* L. (Cucurbitaceae) with accession numbers 50935 and 50934, respectively, were deposited at the herbarium of the Makerere University.

Hydro-ethanol extract of *M*. *charantia’s* immature fruits was prepared according to previously optimized extraction conditions [42]. Briefly, the fruits were cleaned with hypochlorous distilled water, then sliced to remove seeds [43]. The remaining fruit pulps were freeze-dried. The dried pulps were milled to a coarse powder using an electric blender. Then, 50 g of the powder was extracted by maceration with 70% v/v ethanol in the ratio of 1:20. The extraction proceeded for 1 hour at 70°C and 150 rev/m maintained by a shaking water bath (OLS 200, Grants, UK). Immediately after extraction, the mixture was placed on ice to cool. Later, it was filtered first using a muslin cloth and then Whatman filter paper number 1. The solvent was removed from the filtrate under vacuum at 40°C using a rotary evaporator (RV 10D S99, IKA, German). The residue was lyophilized and percentage yield calculated. The dried extract was placed in an airtight container under 4 ± 2°C until use. Aqueous extraction of immature fruits of *A*. *esculentus* was prepared using our previously optimized extraction parameters [44]. Then, the percentage yield was calculated, and a wholly dried extract was weighed and stored in a moisture-free, airtight plastic container, sealed, and kept under 4 ± 2°C until use. The subsequent experiments used *M*. *charantia* fruit extract (MCFE) and *A*. *esculentus* fruit extract (AEFE).

### Physicochemical properties of the extracts

Evaluation of organoleptic features of the extracts was performed by the organ of senses as proposed by the World Health Organization [45]. The dried extracts were assessed individually for appearance, color, odor, and taste. Similarly, the moisture content of the extracts was analyzed using an automatic moisture content analyzer (MJ33, Switzerland). The analyzer dried materials at a temperature of 105°C to a constant weight and then the percentage moisture content was recorded.

The angle of repose (θ) determines powder materials’ flow property [46]. Frequently, the fixed funnel method is used for determining the angle of repose [47]. Briefly, the funnel was positioned at 5 cm above the flat surface. About 10 g of extract powder was poured and retained in the funnel and then allowed to flow through it. After a complete flow, the height and radius of the mound were measured with a ruler. This procedure was performed in triplicate. Finally, the average angle of repose was calculated.

### Extract-extract compatibility

The HPLC method determined the compatibility of the binary mixture of extracts. First, the HPLC chromatogram was analyzed to detect the changes in the number of peaks. Then, the number of peaks of the combined extract was compared with the number of peaks observed in individual extracts. The HPLC chromatogram was developed according to the previous method [44]. Briefly, the chromatographic system consisted of a Shimadzu LC-10AT equipped with an SPD-20A UV/VIS detector (Tokyo, Japan), communicator CBM-20A (Tokyo, Japan), and degassing unit DGU-20A5R (USA) with an isocratic binary system of the mobile phase. Chromatographic separation was performed on a Lunar® C18 column (5 μm; 250 x 4.6 mm; Phenomenex, USA) maintained at a temperature of 40°C in a Shimadzu column oven (CTO-20AC, Tokyo Japan). The mobile phase was methanol: water containing 0.01% trifluoroacetic acid: acetonitrile (60:30:10 v/v), a flow rate of 1 mL/min with a detection wavelength of 210 nm. About 10 μL was injected at a pressure of 2,596.17 psi. The sample was prepared in triplicate using methanol at a 1 mg/mL concentration. For the compatibility study, the binary mixture of the two extracts was prepared in the ratio of 1:1 for analysis. Samples and mobile phases were filtered through 0.45 μm membrane filters (EZ-Pak®, France) before loading into the system.

### Dose of a binary mixture of *A*. *esculentus* and *M*. *charantia for glycemic control*

Response surface methodology (RSM) with a five-level two-factor central composite rotatable design (CCRD) was employed to determine the optimal dose combination of MCFE and AEFE for adequate glycemic control. The design had nine runs. The number of runs comprised of; 4 factorial, 4 axial, and 1 replicate run at the center. The runs were calculated based on Eq 1 [48].


$$N=2^{n}+2n+Nc$$ (1)

Where N = Total number of experimental runs; n = Number of variables; Nc = Replicate runs

The independent factors were concentrations of *M*. *charantia* fruit extract (MCFE) and *A*. *esculentus* fruit extract (AEFE), while the dependent factor was fasting plasma glucose levels (mmol/L). The minimum and maximum doses for MCFE (100–250 mg/kg) were determined based on previous studies [13], while previous optimization experiments determined the dose range of AEFE at 100–250 mg/kg [44]. The experiments were grouped in one block with the factorial and axial points that run one replicate at the center. The distance from the center (α) determines the extreme points, i.e., the lowest and highest values of the factors. For example, the CCRD with two factors, the α is 1.41421. Table 1 shows the actual and coded independent factors.

**Table 1:** Design summary for independent variables.

Factors	Name (mg)	Type	Min	Max	Coded low	Coded high	Mean	SD
**A**	**AEFE**	Numeric	68.93	281.07	-1 ↔ 100	+1 ↔ 250	175	75
**B**	**MCFE**	Numeric	68.93	281.07	-1 ↔ 100	+1 ↔ 250	175	75

AEFE = *Abelmoschus esculentus* fruit extract; MCFE = *Momordica charantia* fruit extract; SD = Standard deviation

#### *In vivo* experimental design for a binary mixture of extracts

*Animal groups*. The experimental design assistant tool (EDA) was employed to design a randomized controlled *in vivo* study with 11 experimental groups (https://eda.nc3rs.org.uk/). The groups (G1-G9) were given test extracts with different doses, G10 was a vehicle control, and G11 was a standard control group. **Animals:** outbred male Wistar albino rats, 15 to 18 weeks old, weighing 240 to 265 g, were obtained from the animal research facility of Mbarara University of Science and Technology. Five animals were housed per cage and acclimatized for seven days with access to a chow diet and water *ad libitum*. The animals were maintained at 24°C ± 2°C and 55% ± 5% humidity, with a 12-h light-dark cycle.

*Sample size determination*. The sample size was calculated based on 0.90 power, 0.789 variabilities (estimated from pilot data), a two-sided unpaired t-test at a 0.05 significant level, and a unit desired change in FPG. In addition, the vehicle control group’s sample size was adjusted according to Bate’s method to improve the study’s sensitivity [49]. The sample size adjustment was necessary because the factorial experiment involved multiple test groups and only one vehicle control group. Thus, the final sample size was 65 animals, with each test group having five animals while the vehicle control group had ten animals.

*Induction of prediabetes*. Prediabetes was induced by feeding animals a high-fat diet (HFD) for eight weeks [50]. The HFD with 50% energy as fat and the control diet with 10% energy as fat were prepared in-house based on previous diets [51]. The pure ingredients were formed into a dough and stored at 4 ± 2°C to prevent rancidity. Fresh diets were prepared biweekly.

*Treatment*. The AEFE and MCFE extracts were separately dissolved in distilled water. The appropriate volume of each extract was mixed shortly before administering it to animals. The volume of a mixture of the extract was not more than 2 mL/100 g. Then, the mixed extract was given to G1-G9 by oral gavage. The vehicle control group (G11) received distilled water at a volume of 2 mL/100 g, and the standard diabetic control group (G10) received glibenclamide 5 mg/kg. The test extracts, distilled water, and standard drug were given daily for 21 days. During treatment, animals continued with their diets, and every seven days, FPG and body weight were measured. After the last measurement, animals were humanely sacrificed.

*Outcome measurements*. The primary outcome -the FPG, was measured from the rat’s tail blood using the Accu-Chek® Active (Roche, Mannheim, Germany) glucometer in mmol/L. Before each measurement, the animals fasted for 8 h to reduce FPG levels’ variability [52]. The secondary outcome–the fasting body weight (g)- was recorded using a digital weighing balance at five different time points. At the end of the study period (21 days), the animals were euthanized by inhaling 4% halothane in a closed chamber. Death was confirmed by lack of pulse, breathing, and response to a firm toe pinch. Then, dead animals were disposed of correctly.

### Screening of excipients

Potential excipients were screened in a set of four experiments (Table 2). In these experiments, various concentrations and types of excipients were tested to identify their effect on the granules’ technological properties, including flowability measured by the Hausner ratio (HR), bulky density, and tapped density [46]. The excipients that resulted in ‘excellent’ flow indicated by HR of < 1.11 or good flow by HR of 1.12–1.18 were selected for subsequent formulation development [53].

**Table 2:** Proposed formulation compositions for herbal capsule.

Component	F1	F2	F3	F4
**MCFE**	X mg	X mg	X mg	X mg
**AEFE**	Y mg	Y mg	Y mg	Y mg
**Magnesium carbonate (%)**	16.67	-	16.67	15.00
**Lactose (%)**	-	16.67	-	-
**Talc (%)**	1.67	-	0.50	0.50
**Magnesium stearate (%)**	5.00	5.00	-	0.17
**Sodium hydrogen carbonate (%)**	0.57	2.20	2.20	2.20
**Sodium benzoate (%)**	0.10	0.10	0.10	0.10
**Quantity per capsule (mg)**	600	600	600	600

F = Formulation; MCFE = *Momordica charantia* fruit extract; AEFE = *Abelmoschus esculentus* fruit extract

The bulk density and tapped density were determined by taking 30 g of granules and placed in a 100 mL graduated measuring cylinder. First, the volume occupied by the granules was noted as bulk volume. The measuring cylinder was then tapped from 5 cm height until no apparent volume change was noted. Then, the volume was re-recorded as tapped volume [53]. Next, the bulk density (ρ_b_) and tapped density (ρ_t_) were calculated based on Eqs 2 and 3, respectively [53]. Finally, these values were used to calculate HR based on Eq 4 [53].


$$\rho b=\frac{Weight\;of\;granules\;(g)}{Bulk\;volume\;(mL)}$$ (2)

$$\rho t=\frac{Weight\;of\;granules\;(g)}{Tapped\;volume\;(mL)}$$ (3)

$${Hausner}^{\prime }s\;ratio\;(HR)=\frac{\rho t}{\rho b}$$ (4)

First, the *Momordica charantia* fruit extract (MCFE) was pretreated with talc and a portion of magnesium stearate to reduce stickiness. Next, *Abelmoschus esculentus* fruit extract (AEFE) was blended with magnesium carbonate or lactose, sodium hydrogen carbonate, and sodium benzoate using a mixer for 5 min. Then, the MCFE mixture was combined with the AEFE mixture and dried in an electric oven at 60°C for 50 minutes. Finally, dry granulation was performed by hand sieve through 180 sieves. The granules were mixed with lubricant–the magnesium stearate. The granules were then filled into empty capsule shells of size 00 using a manual capsule filling machine (Lodha, LI-CFM 300, India).

### Formulation development using D-optimal mixture design

A D-optimal mixture design was employed to determine the effects of the selected excipients (independent variables) on granules’ flowability and capsule disintegration time (response variables) [37]. These response variables were considered as herbal capsule formulation’s critical quality attributes (CQA) [54]. Thus, a five-level, four factors D-optimal mixture design afforded twelve experiments with a proportions range of excipients as follows; A: MgCO3 (10.25–12.9%), B: Magnesium stearate (1.0–2.0%), C: Talc (5.0–10.0%), G: Sodium bicarbonate (1.0–5.0%), and D: Sodium benzoate (0.1%). The actual composition of the 600 mg herbal capsule is presented in Table 3. The quantities of the two extracts were determined from previous factorial experiments.

**Table 3:** Actual and coded design constraints for the herbal capsule formulation.

Component (mg)	Min.	Max.	Coded Low	Coded High	Mean	Std. Dev.
**A: MgCO3**	61.40	77.40	+0 ↔ 61.40	+0.44 ↔ 77.40	69.67	6.69
**B: Mg stearate**	6	12	+0 ↔ 6	+0.17 ↔ 12	8.27	2.58
**C: Talc**	30	60	+0 ↔ 30	+0.83 ↔ 60	44.91	9.42
**D: Sodium benzoate**	0.60	0.60	+0 ↔ 0.60	+0 ↔ 0.60	0.60	0.00
**E: MCFE**	X	X	+0 ↔ X	+0 ↔ X	X	SD
**F: AEFE**	Y	Y	+0 ↔ Y	+0 ↔ Y	Y	SD
**G: Sodium bicarbonate**	10	30	+0 ↔ 10	+0.56 ↔ 30	20.55	7.82

X = Amount of MCFE extract, Y = amount of AEFE extract

### Herbal capsule optimization using the D-optimal mixture design

The optimal levels of excipients that meet the criteria for good to excellent granules flowability (HR ≤ 1.18) and short disintegration time (≤ 30 min.) were obtained from overlay graphs. In addition, response surface plots were plotted to study the interactions of several independent variables on granule flowability and capsule disintegration time. Finally, verifying the optimum checkpoints was done by selecting an optimum point with a high desirability factor to prepare the dosage form.

### Herbal capsule formulation evaluation

The herbal capsules were assessed for uniformity of weight, moisture content, disintegration time, and total polyphenols and total flavonoids assays. The British Pharmacopoeia (2000) method was used for uniformity of weight. Briefly, 20 capsules were randomly selected. Each capsule was weighed and then completely emptied of its contents, and the empty capsule weighed. First, the capsule contents’ weight was calculated by subtracting the empty capsule’s weight from the filled capsule’s initial weight. Next, the average values for the 20 capsules and percent deviation were calculated. The capsules pass the test if not more than two of the capsules’ weights deviate from the average weight by more than 7.5%, and none of the deviates by more than twice that percentage.

Loss on drying method involved thermo-balance moisture analyzer (MJ33, Switzerland) determined the moisture content of the capsule’s contents. The capsule’s shell was removed, and its content was placed on an aluminium plate at 105°C in an electrically heated chamber. The unit then automatically weighs moisture loss from the sample. The heating stopped after reaching a constant weight of the sample.

According to International Pharmacopoeia ninth edition 2019 [55], disintegration time was assessed using disintegration apparatus A (DTG 2000, Nottingham, UK). Briefly, one capsule was placed in each of the six tubes of the basket, with the disc to prevent capsules from floating. Distilled water at a temperature of 37°C was used as a fluid medium. As reported in our previous study, total polyphenols and flavonoids were quantified by UV/VS spectrophotometry [44].

The total polyphenols content (TPC) was determined according to the Folin-Ciocalteu reagent method [56]. About 1 mg/mL sample solution was oxidized with 2 mL of 10% v/v Follin-Ciocalteu reagent (FCR) and 2 mL of 7.5% sodium carbonate. The mixture was incubated for 40 minutes at 45°C, and absorbance was measured at 765 nm using a UV/VIS spectrophotometer (JENWAY, 6705; Stone, Staffs, UK). The TPC was calculated as a Gallic acid equivalent.

The capsule’s total flavonoid content (TFC) was determined [57]. Briefly, 1 mg/mL of crude extract was mixed with 4 mL of distilled water and 0.5 mL of 5% NaNO2, then allowed to stand for 5 minutes. Later, 0.5 mL of 10% AlCl_3_ was added and allowed to stand for 6 minutes before adding 1 mL of 1 M NaOH and making the mixture 10 mL with distilled water. Finally, the mixture was allowed to stand for 15 minutes at room temperature, and absorbance was measured at 510 nm using a UV/VIS spectrophotometer (JENWAY, 6705; Stone, Staffs, UK). The TFC was calculated as quercetin equivalent based on the standard calibration curve.

#### Data management and analysis

The analysis was done by Design-expert^®^ software version 12 (Stat-Ease Inc., Minneapolis, MN, USA). Then data were fitted in a higher-order quadratic polynomial model. The adequacy of the model was determined by assessing the *p*-value of the lack of fit, coefficient of determination (*R*^2^), and ANOVA F-value. Finally, the three-dimensional response surface and overlay graphs were plotted for optimal formulation.

#### Ethical considerations

Mbarara University of science and technology research ethical committee (MUST-REC) approved the study protocol (MUST-REC 19/01-19). Also, the protocol was approved by the Uganda National Council for Science and Technology (UNCST) with research registration number NS119ES.

## Results

### Plant extracts and physicochemical properties

The dried extracts of fruits of *M*. *charantia* and *A*. *esculentus* were labeled as MCFE and AEFE, respectively. The MCFE had a percentage yield of 19.8% lower than that of AEFE 26.21%. Assessment of color and physical appearance indicated that the MCFE was bitter, dark brown, sticky gum-like, and characteristic smell, while AEFE was a tasteless, yellowish-brown, free-flowing, and fruity smell. The moisture content of each dried extract was estimated in triplicate. The average percentage of moisture content for AEFE and MCFE was 4.49 ± 0.19 and 5.71 ± 0.38%, respectively. In addition, the estimation of the angle of repose for each extract indicated that the AEFE has an angle of repose of 33.69°, which means AEFE powder has a ‘good’ flow property (European pharmacopeia). Conversely, the MEFE is sticky and does not freely flow.

### Compatibility of plant extracts

**Fig 1:**
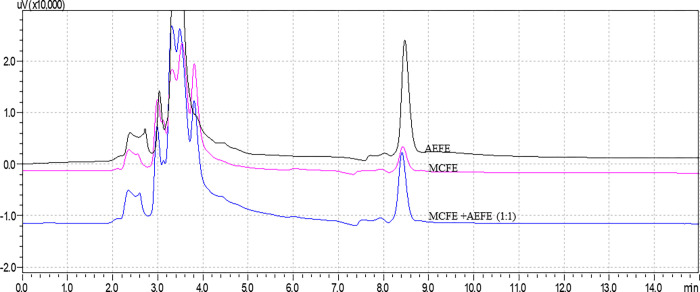
Extract-extract compatibility profile. The black, purple, and blue chromatograms represent chemical fingerprints for AEFE, MCFE, and their binary mixture (1:1). A well-resolved peak was found at approximately 8.5 minutes in all the preparations. Conditions: mobile phase: 0.01% trifluoroacetic acid water: methanol: acetonitrile (30:60:10 v/v); Column C18; Detection: 210 nm.

The chemical fingerprints of the binary mixture of the two extracts (MCFE and AEFE) in a 1:1 ratio were developed using an HPLC technique. Qualitative analysis of the chromatogram peak size and number indicated that the combined extract retained peak numbers and patterns observed in the individual extracts (Fig 1 and S1 Table). In addition, a shared, well-resolved peak was detected at approximately 8.5 min retention time. Although it occurred in both extracts, this diagnostic peak was more prominent in extract AEFE than MCFE and combined extract suggesting the lack of potential chemical interaction. Although there was some variation in peak number, that is the AEFE chromatogram had 14 peaks and MCFE had 15 peaks, the combined extract retains the peak patterns observed in individual extracts with 12 peaks implying the absence of significant chemical interactions (Fig 1 and S1 Table). This preliminary qualitative investigation signifies that the two extracts could be combined in a dual herbal dosage form.

### Dose of combined extract

There was a significant difference among the 11 treatment groups on mean FPG levels at day 7, day 14, and day 21 of follow up, F (10, 54) = 3.547, *p* = 0.002, F (10, 54) = 6.489, *p* <0.001, F (10, 54) = 7.836, *p* < 0.001, respectively (Table 4). Multiple comparison tests on day 21 indicated a significant difference in mean FPG levels of G1, G2, G5, G6, G8, and G10 compared to G11 *p* <0.05. Among these groups, group G5 had a large mean difference at -2.4. Therefore, the dose of a binary mixture of fruit extracts given to G5 was considered in the subsequent formulation development.

**Table 4:** Effect of varied dose ratio on fasting plasma glucose levels in rats.

Groups ID	Dose (MCFE + AEFE) mg/kg	Fasting Plasma glucose (Mean ± SEM) (mmol/L)			
		Baseline	7 days^#^	14 days*	21 days*
**G1**	175 + 68.90	6.2 ± 0.25	5.8 ± 0.24	4.6 ± 0.18	4.6 ± 0.20^$^
**G2**	100 + 100	6.0 ± 0.12	5.8 ± 0.21	4.9 ± 0.21	4.8 ± 0.18^$^
**G3**	68.9 + 175	6.2 ± 0.38	6.1 ± 0.39	5.4 ± 0.53	5.5 ± 0.35
**G4**	250 + 100	6.3 ± 0.30	6.0 ± 0.17	5.1 ± 0.23	5.3 ± 0.29
**G5**	175 + 281	6.4 ± 0.43	4.9 ± 0.27	3.9 ± 0.13	4.1 ± 0.07^$^
**G6**	250 + 250	6.2 ± 0.15	5.7 ± 0.17	5.2 ± 0.17	5.0 ± 0.19^$^
**G7**	175 + 175	6.5 ± 0.26	6.0 ± 0.10	5.4 ± 0.17	5.3 ± 0.27
**G8**	100 + 250	6.1 ± 0.08	5.8 ± 0.30	5.1 ± 0.21	5.0 ± 0.18^$^
**G9**	281+ 175	6.4 ± 0.28	5.9 ± 0.12	5.5 ± 0.32	5.3 ± 0.25
**G10**	Glibenclamide (5 mg/kg)	6.3 ± 0.28	5.9 ± 0.24	5.0 ± 0.29	4.8 ± 0.12^$^
**G11**	Distilled water (2 mL/100g)	6.5 ± 0.20	6.7 ± 0.07	6.6 ± 0.17	6.5 ± 0.08

n = 5

^#^*P* = 0.002

*P <0.001 ANOVA test

^$^*P* = <0.05 Games Hawell multiple comparison test

The varied ratio of AEFE and MCFE had no statistically significant effect on mean body weight between groups at baseline, 7, 14, and 21 days of treatment, *p* > 0.05 (S2 Table).

### Excipients selection

The excipients screening stage involved assessing the influence of excipients on granules’ properties. According to Table 5, formulations F3 and F4 had excellent flow properties as indicated by Hausner’s ratio of < 1.11, whereas F1 and F2 had a ‘good’ and ‘fair’ flow, respectively. The F3, F4, and F1 had magnesium carbonate as a filler that proved to contribute to their excellent to a good flow. Furthermore, the powder density of the granules with the excellent flow was noted as 0.8451 g/mL. Conversely, F2 had lactose as a filler that seems to impact granules flow properties unfavorably. Lactose also seems to retain more water in the granules than its magnesium carbonate counterpart. Hence, lactose was dropped in subsequent formulation design experiments.

**Table 5:** Technological properties of proposed herbal capsule formulations.

Property	F1	F2	F3	F4
**MC %**	3.7 ± 1.12	5.1 ± 1.81	3.2 ± 1.03	3.4 ± 1.46
**Tapped density (g/mL)**	0.79	0.99	0.86	0.92
**Bulk density (g/mL)**	0.68	0.83	0.85	0.83
**HR**	1.17	1.20	1.02	1.09

MC = Moisture content, HR = Hausner ratio, F = Formulation.

### D-optimal mixture design

The D-optimal mixture design provided 12 experiments on the effects of different amounts of excipients: magnesium carbonate (A), magnesium stearate (B), talc (C), sodium benzoate (D), sodium bicarbonate (G), and active ingredient (mixture of MCFE and AEFE) on granules flowability and capsule disintegration time responses (Table 6).

**Table 6:** The compositions and quality attributes of herbal capsule formulation.

Run	Amount measured in mg							HR	Disintegration time (min.)
	A	B	C	D	*E*	*F*	G		
**1**	61.40	6.00	60.00	0.60	*175*	*281*	16.00	3.14	50
**2**	62.70	8.50	42.20	0.60	*175*	*281*	30.00	1.20	25
**3**	76.80	9.10	37.00	0.60	*175*	*281*	20.40	1.13	25
**4**	77.40	12.00	44.00	0.60	*175*	*281*	10.00	1.90	55
**5**	61.40	6.00	50.80	0.60	*175*	*281*	25.20	2.19	30
**6**	77.40	6.00	50.00	0.60	*175*	*281*	10.00	1.52	50
**7**	67.80	9.30	56.2.	0.60	*175*	*281*	10.00	1.27	35
**8**	70.90	6.00	38.80	0.60	*175*	*281*	27.70	1.16	25
**9**	71.40	12.00	31.80	0.60	*175*	*281*	28.20	2.13	30
**10**	77.40	6.00	30.00	0.60	*175*	*281*	30.00	2.77	30
**11**	69.90	6.40	45.60	0.60	*175*	*281*	21.50	1.02	20
**12**	61.40	12.00	52.40	0.60	*175*	*281*	17.60	2.98	25

A = Magnesium carbonate; B = Magnesium stearate; C = Talc; D = Sodium benzoate; E = MCFE; F = AEFE; G = Sodium bicarbonate; HR = Hausner ratio, Dist. Time = Disintegration time (minutes).

**Fig 2:**
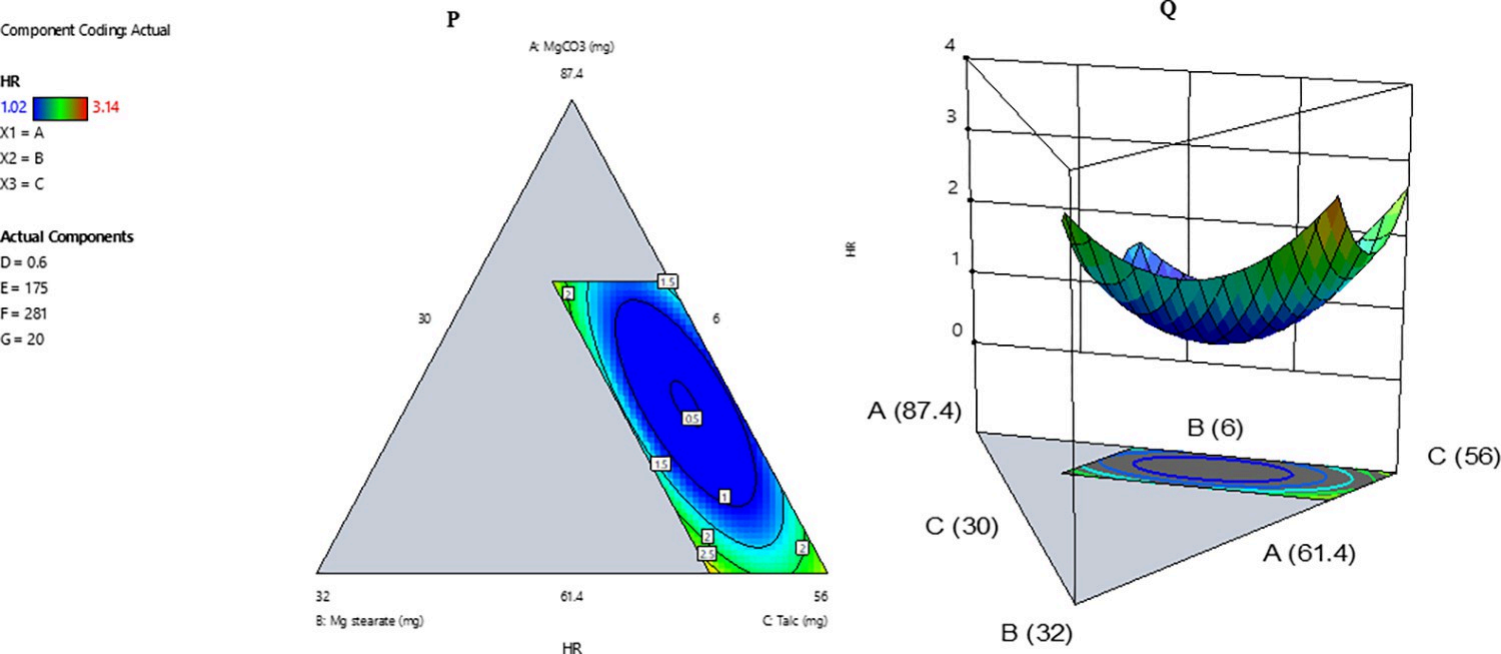
Model contour presentation. The contour diagram (P) and the 3D diagram (Q) represent three-component systems. It shows the relationships between the levels of the independent variables (magnesium carbonate (A), magnesium stearate (B), and talc (C) at a fixed level of sodium bicarbonate (G) of 20 mg, Sodium benzoate (D) of 0.6 mg, and 76% of active pharmaceutical ingredients (E = MCFE and F = AEFE) on flowability as measured by Hausner’s ratio (HR). Each tip of the triangle indicates each independent variable’s maximum level, whereas the three lines (AB, BC, CA) represent a binary mixture with the third variable kept on a fixed level in the center of the line. The blue-colored space indicates the combinations where the best desirable flowability can be achieved. The decrease in the amount of A and B increases flowability (low HR), with further decrease, leading to a decrease in flowability (high HR).

The granules flowability analysis (response variable) showed a statistically significant quadratic model fitting (Table 7). The ANOVA results indicated the model F-value of 59.87 implies the model is significant (*p* = 0.0165). There is only a 1.65% chance that an F-value this large could occur due to noise. The linear terms and all second-order interaction terms were significant (*P* < 0.05) (Table 7). The adjusted R-squared of 0.9797 was greater than 0.75, suggesting that the number of model terms adequately fit the model with great precision. The mathematical model Eq 5 and the contour presentation (Fig 2) are below.

**Table 7:** ANOVA for a quadratic model for flowability.

Source	Sum of Squares	df	Mean Square	F-value	*p*-value
Model	6.51	9	0.70	59.87	0.0165*
⁽^1^⁾Linear Mixture	0.75	3	0.25	20.55	0.0468*
AB	1.60	1	1.60	132.57	0.0075*
AC	2.11	1	2.11	174.53	0.0057*
AG	0.57	1	0.57	47.16	0.0206*
BC	1.31	1	1.33	108.59	0.0091*
BG	1.19	1	1.19	98.39	0.0100*
CG	0.28	1	0.28	22.92	0.0410*
Residual	0.02	2	0.01		
Cor Total	6.53	11			

df = degree of freedom; A = Magnesium carbonate; B = Magnesium stearate; C = Talc; G = Sodium bicarbonate.

$Hausner^{\prime }s\;Ratio=10.12\times A+101.33\times B+4.15\times C+2.73\times G-131.91\times AB-21.35\times AC-13.3\times AG-118.12\times BC-115.87\times BG-5.54\times CG$        (5)

Conversely, the quadratic model for disintegration time was not statistically significant (*p* = 0.1272). Thus, the mathematical model equation was not adopted.

### Optimization of granules flowability for herbal capsules formulation

**Fig 3:**
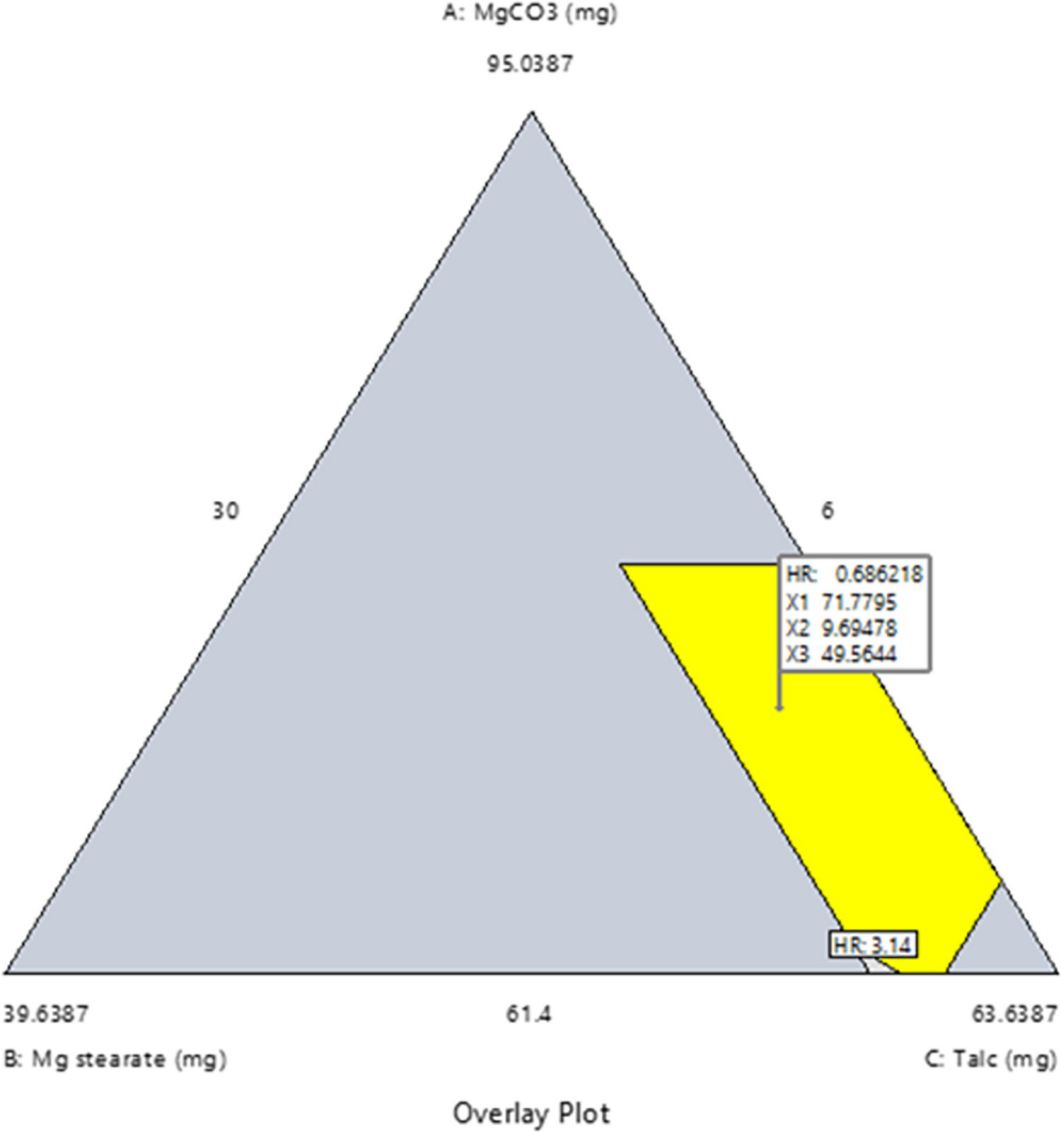
Overlay plot for optimal levels of excipients in a capsule formulation. The overlay plot illustrates the design space of the effects of magnesium carbonate (A = X_1_), magnesium stearate (B = X_2_), talc (C = X_3_) at a fixed level of sodium bicarbonate (G) of 12.3613, and 76% of active pharmaceutical ingredients (MCFE and AEFE) on flowability measured by Hausner’s ratio. The yellow areas (design space) indicate the region where excellent flowability can be obtained.

The optimal points from the design space for the herbal capsule dosage form (Fig 3) were used in a confirmation experiment.

### Confirmation of optimized formulation compositions

The confirmation experiments were performed in triplicate, and the findings showed that the actual values of flowability and disintegration time were within the 95% confidence interval (Table 8). These findings imply that the model successfully predicted the capsule’s critical quality attributes.

**Table 8:** Results of confirmation experiments.

Response	Predicted Mean	SE Pred.	Observed mean	n	95% CI	
Flowability	0.69	0.10	0.83	3	0.24	1.13
Disintegration time (Min)	29.99	4.47	26.67	3	10.75	39.23

### Evaluation of capsules quality attributes

**Fig 4:**
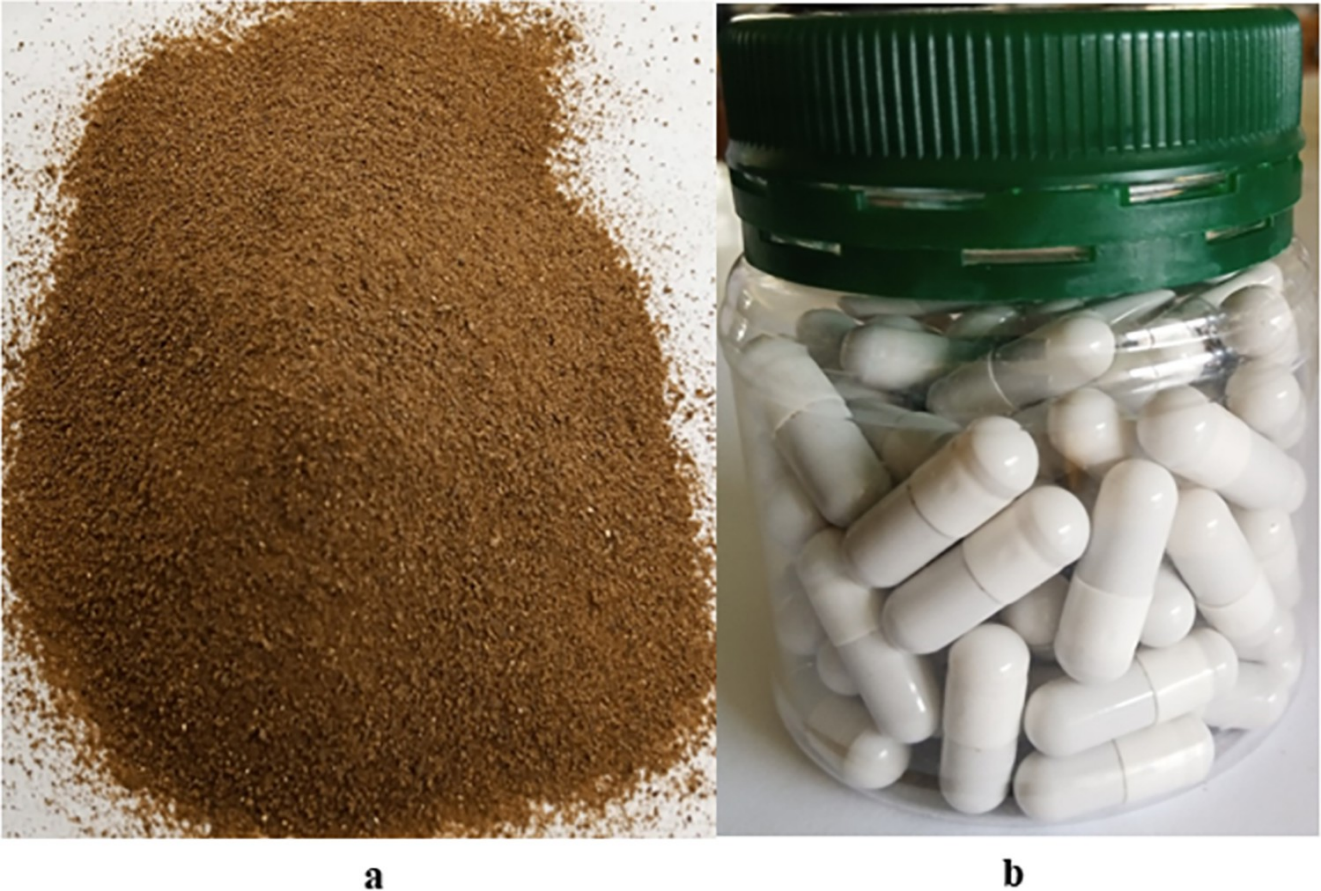
Herbal granules (A) and the DM083 capsules (B). (**a**) Herbal granules contain 76% of dried herbal extracts as active pharmaceutical ingredients mixed with excipients. (**b**) Herbal capsules in a plastic airtight container; were produced using a manual capsule filling machine [Lodha, LI-CFM 300, India]. The capsules have a size of 00 and contain 600 mg of herbal granules.

The final optimized granules are brownish, free-flowing, with a sweet smell (Fig 4A). The resultant standardized 600 mg herbal capsule dosage form containing 76.0% dry herbal extract was named DM083 (Fig 4B). The capsules have an acceptable pharmaceutical appearance (clean with no dust adhered, shiny, tightly locked, and no deformation of the gelatin shell), effectively mask the herbal materials’ bitter taste, off-white colored, and are odorless.

The average deviation in weight from the average DM083 capsule weight is 1.4 ± 0.96% (S3 Table). The British Pharmacopoeia 2000 prescribed the acceptable limit of ± 7.5% of the average capsule weight. Thus, our findings indicate that the DM083 capsules meet the quality requirement set by British Pharmacopoeia 2000. Moreover, the capsules had an average moisture content of 4.25 ± 0.247% and a disintegration time of 26.6 ± 4.75 minutes.

The TPC of the DM083 capsule was estimated based on a standard calibration curve with an *R*^*2*^ of 0.9923. The determination of TPC was done in triplicates. The results showed that DM083 had a mean TPC of 40.41 ± 0.62 mg GAE/g DW. While, the TFC calculated based on the calibration curve with an *R*^*2*^ = 0.9966, indicated that the DM083 had an average TFC of 25.5 ± 0.38 mg QE/100g DW.

## Discussion

The D-optimal mixture design provided a model that predicted the effect of formulation components on the capsule’s critical quality attributes. Ultimately, the 600 mg herbal capsules with 76% drug loading were developed. This study reports for the first time such a standardized herbal capsule containing a mixture of *M*. *charantia* and *A*. *esculentu*s dried fruit extracts. These findings have important implications in addressing the inherent shortfalls of traditional liquid dosage forms. The herbal capsules (DM083) can provide a consistent dose, ensure dose uniformity among users, improve shelf life, reduce bulkiness, and are convenient for patients. Additionally, the findings support the hypothesis that extracts from the two plant species could be combined into a stable solid dosage form.

The D-optimal mixture design is the most popular response surface methodology used in formulation studies [37–39]. It investigates the main effects and interaction effects between the independent variables of the experiment on the responses. Since, in formulation development, the responses of interest are a function of the composition of the mixture, With D-optimal mixture design, the interactions of the mixture’s composition could be modeled, graphically or mathematically, to reveal regions of desirable formulation compositions that meet the product’s quality criteria. Our findings that the D-optimal mixture approach provides a model that adequately predicted the optimal design space are similar to those reported in other studies [37–39, 58].

The unfavorable physicochemical properties of MCFE and AEFE, such as hygroscopicity and stickiness, cause poor granule flowability and prolong disintegration time. Therefore, the use of magnesium stearate as glidant/lubricant, magnesium carbonate as a filler and absorbent, and sodium hydrogen carbonate as disintegrating aiding agents significantly improved the capsule’s critical quality attributes (granules flowability and disintegration time).

Our findings that *M*. *charantia* could be combined with other plant species as a therapeutic strategy to manage T2DM agree with previous studies that developed polyherbal formulations using *M*. *charantia*. For example, Suthar et al., 2016 used a patented juice preparation procedure (not disclosed) to make juice using whole fruits of *M*. *charantia*. Then developed PDM011011-capsule contained 400 mg of dry standardized fruit juice powder with not less than 0.4 mg of Uridine as a chemical marker [59]. Similarly, an Ayurvedic formulation GlucoCare capsules manufactured by The Himalaya Drug Company, Bengaluru, India, used *M*. *charantia* fruit extract combined with 22 extracts from different plant species [60]. In another study, US Botanicals (Bell Gardens, CA) manufactured herbal capsules containing *M*. *charantia* seeds mixed with ten herbal extracts to form a pancreas tonic [61]. However, these herbal formulations had limited standardization details and lacked quality control aspects of the formulation.

One of the limitations of this study is that plant raw materials studied were obtained from a supplier that did not follow the guideline for good agricultural and collection practices for herbal materials as recommended by the WHO [62]. Therefore, using such herbal materials to formulate herbal medicine could have introduced substandard materials and compromised quality. However, efforts were made to ensure that inferior quality herbal materials were sorted, and only those with uniform quality were processed.

## Conclusion

The D-optimal mixture design was successfully applied to formulate an herbal capsule dosage form with a high drug loading of 76%. The optimized herbal formulation met the acceptable critical quality attributes requirements, having a disintegration time of 26.6 ± 4.75 minutes, excellent granules flowability (Hausner ratio < 1.11), TPC of 40.41±0.62 mg GAE/gDW, and TFC of 25.5±0.38 mg QE/gDW as drug content. Further biological studies such as toxicity and long-term efficacy studies could be carried out before large-scale commercial production.

## Supporting information

S1 Table Peak table. (DOCX)

S2 Table Effect of varying doses on the bodyweight. (DOCX)

S3 Table Weight variation of DM083 capsules. (DOCX)
