# Supplementary material for: D-optimal mixture design optimized solid formulation containing fruits extracts of Momordica charantia and Abelmoschus esculentus
Source: PLoS One. 2022 Jun 24;17(6):e0270547. doi: 10.1371/journal.pone.0270547 (PMC9232165; doi:10.1371/journal.pone.0270547)
Supplement: S1 Table — (DOCX) [file pone.0270547.s001.docx]

**Table 1. Peak table for AEFE, MCFE and their binary mixture**

| **AEFE** |  |  |  |  |  |  |
| --- | --- | --- | --- | --- | --- | --- |
| **Peak#** | **Ret. Time** | **Area** | **Height** | **Peak Start** | **Peak End** | **Area%** |
| **1** | 2.384 | 112622 | 5547 | 0.017 | 2.575 | 5.5505 |
| **2** | 2.711 | 68687 | 6257 | 2.575 | 2.867 | 3.3852 |
| **3** | 3.030 | 111063 | 13418 | 2.867 | 3.142 | 5.4737 |
| **4** | 3.357 | 580817 | 46185 | 3.142 | 3.483 | 28.6252 |
| **5** | 3.517 | 557775 | 38266 | 3.483 | 4.300 | 27.4896 |
| **6** | 4.333 | 16698 | 3074 | 4.300 | 4.392 | 0.8230 |
| **7** | 4.454 | 78424 | 3084 | 4.392 | 5.200 | 3.8651 |
| **8** | 5.275 | 8294 | 553 | 5.200 | 5.475 | 0.4088 |
| **9** | 5.558 | 8260 | 441 | 5.475 | 6.150 | 0.4071 |
| **10** | 7.002 | 3565 | 99 | 6.933 | 7.550 | 0.1757 |
| **11** | 7.715 | 11165 | 985 | 7.583 | 7.833 | 0.5503 |
| **12** | 8.017 | 22193 | 1366 | 7.833 | 8.167 | 1.0938 |
| **13** | 8.468 | 310281 | 23239 | 8.167 | 8.850 | 15.2920 |
| **14** | 9.145 | 139195 | 1493 | 8.850 | 12.200 | 6.8601 |
| **MCFE** |  |  |  |  |  |  |
| **1** | 2.103 | 3874 | 405 | 1.858 | 2.167 | 0.2848 |
| **2** | 2.358 | 56952 | 3987 | 2.167 | 2.517 | 4.1871 |
| **3** | 2.550 | 28562 | 3214 | 2.517 | 2.775 | 2.0998 |
| **4** | 2.990 | 128610 | 13339 | 2.775 | 3.083 | 9.4554 |
| **5** | 3.112 | 55680 | 9512 | 3.083 | 3.192 | 4.0936 |
| **6** | 3.312 | 206080 | 18891 | 3.192 | 3.408 | 15.1510 |
| **7** | 3.532 | 333949 | 23676 | 3.408 | 3.708 | 24.5518 |
| **8** | 3.802 | 423007 | 19969 | 3.708 | 7.325 | 31.0993 |
| **9** | 6.031 | 9505 | 398 | 5.800 | 6.400 | 0.6988 |
| **10** | 6.492 | 6906 | 356 | 6.400 | 6.733 | 0.5078 |
| **11** | 6.883 | 9252 | 415 | 6.733 | 7.300 | 0.6802 |
| **12** | 7.581 | 11773 | 628 | 7.333 | 7.725 | 0.8656 |
| **13** | 7.934 | 13607 | 795 | 7.725 | 8.125 | 1.0004 |
| **14** | 8.424 | 67473 | 4832 | 8.125 | 8.733 | 4.9606 |
| **15** | 8.823 | 4949 | 368 | 8.733 | 9.175 | 0.3638 |
| **mixture** |  |  |  |  |  |  |
| **1** | 2.113 | 6529 | 656 | 1.792 | 2.167 | 0.3904 |
| **2** | 2.348 | 66490 | 4522 | 2.167 | 2.508 | 3.9758 |
| **3** | 2.591 | 42514 | 4177 | 2.508 | 2.783 | 2.5422 |
| **4** | 2.979 | 124634 | 13275 | 2.783 | 3.083 | 7.4526 |
| **5** | 3.121 | 41833 | 8596 | 3.083 | 3.167 | 2.5015 |
| **6** | 3.303 | 306358 | 26921 | 3.167 | 3.408 | 18.3190 |
| **7** | 3.483 | 369059 | 26527 | 3.408 | 3.708 | 22.0683 |
| **8** | 3.799 | 555530 | 16750 | 3.708 | 7.383 | 33.2185 |
| **9** | 5.997 | 1991 | 150 | 5.883 | 6.358 | 0.1191 |
| **10** | 7.561 | 11807 | 751 | 7.383 | 7.725 | 0.7060 |
| **11** | 7.925 | 15340 | 913 | 7.725 | 8.108 | 0.9173 |
| **12** | 8.405 | 130265 | 9739 | 8.108 | 9.200 | 7.7893 |
