# Supplementary material for: D-optimal mixture design optimized solid formulation containing fruits extracts of Momordica charantia and Abelmoschus esculentus
Source: PLoS One. 2022 Jun 24;17(6):e0270547. doi: 10.1371/journal.pone.0270547 (PMC9232165; doi:10.1371/journal.pone.0270547)
Supplement: S2 Table — (DOCX) [file pone.0270547.s002.docx]

**Table 2. Effect of varied dose ratio of extracts on rat’s fasting body weight**

| **Groups ID** | **Dose (MCFE + AEFE) mg** | **Bodyweight (Mean ± SEM) (g)** | | | |
| --- | --- | --- | --- | --- | --- |
|  |  | **Baseline** | **7 days** | **14 days** | **21 days** |
| **G1** | 175 + 68.9 | 275.0 ±4.59 | 230.2 ± 27.90 | 231.2 ±30.19 | 238.8 ±27.14 |
| **G2** | 100 + 100 | 267.6 ±9.81 | 232.8 ± 18.95 | 227.8 ±20.79 | 231.2 ±20.72 |
| **G3** | 68.9 + 175 | 259.6±14.62 | 222.8 ± 20.26 | 218.0 ±20.39 | 232.4 ±20.75 |
| **G4** | 250 + 100 | 257.0±14.60 | 227.4 ± 19.68 | 222.2 ±19.83 | 232.4 ±20.48 |
| **G5** | 175 + 281 | 261.6 ±23.48 | 239.8 ± 28.71 | 244.0 ±26.36 | 259.6 ±21.16 |
| **G6** | 250 + 250 | 258.6 ±19.18 | 260.2 ± 16.46 | 258.2 ±15.70 | 262.4 ±14.66 |
| **G7** | 175 + 175 | 235.2 ±17.76 | 243.4 ± 16.24 | 249.6 ±17.56 | 252.8 ±17.26 |
| **G8** | 100 + 250 | 257.6 ±10.78 | 266.2 ± 10.13 | 265.0 ±10.82 | 268.8 ±8.49 |
| **G9** | 281+ 175 | 258.2 ±15.57 | 262.0 ± 11.99 | 267.2 ±13.18 | 270.6 ±11.27 |
| **G10** | Glib. | 267.2 ±11.82 | 275.6 ± 11.50 | 278.0 ±8.76 | 273.8 ±7.81 |
| **G11** | DW | 278.4 ±9.88 | 276.0 ± 7.91 | 280.0 ±7.64 | 280.2 ±8.06 |

*p* > 0.05; DW = Distilled water (2 mL/100g); Glib = Glibenclamide (5 mg/kg); n = 5
