# Supplementary material for: D-optimal mixture design optimized solid formulation containing fruits extracts of Momordica charantia and Abelmoschus esculentus
Source: PLoS One. 2022 Jun 24;17(6):e0270547. doi: 10.1371/journal.pone.0270547 (PMC9232165; doi:10.1371/journal.pone.0270547)
Supplement: S3 Table — (DOCX) [file pone.0270547.s003.docx]

**Table 3. Weight variation of DM083 capsules**

| **Capsule** | **W1 (g)** | **W2 g)** | **W3 = (W1-W2)** | **Deviation**  **(Average -W3)** | **%Deviation** |
| --- | --- | --- | --- | --- | --- |
| **1** | 0.8205 | 0.1333 | 0.6872 | 0.01 | 1.43 |
| **2** | 0.7926 | 0.1294 | 0.6632 | 0.01 | 2.12 |
| **3** | 0.7991 | 0.1269 | 0.6722 | 0.01 | 0.79 |
| **4** | 0.8022 | 0.1266 | 0.6756 | 0.00 | 0.28 |
| **5** | 0.787 | 0.1264 | 0.6606 | 0.02 | 2.50 |
| **6** | 0.7875 | 0.1287 | 0.6588 | 0.02 | 2.76 |
| **7** | 0.8066 | 0.1258 | 0.6808 | 0.00 | 0.48 |
| **8** | 0.8126 | 0.1247 | 0.6879 | 0.01 | 1.53 |
| **9** | 0.7973 | 0.1268 | 0.6705 | 0.01 | 1.04 |
| **10** | 0.802 | 0.1274 | 0.6746 | 0.00 | 0.43 |
| **11** | 0.7954 | 0.127 | 0.6684 | 0.01 | 1.35 |
| **12** | 0.8171 | 0.1291 | 0.6880 | 0.01 | 1.55 |
| **13** | 0.8174 | 0.1257 | 0.6917 | 0.01 | 2.09 |
| **14** | 0.8244 | 0.1249 | 0.6995 | 0.02 | 3.24 |
| **15** | 0.7916 | 0.1288 | 0.6628 | 0.01 | 2.17 |
| **16** | 0.8243 | 0.13 | 0.6943 | 0.02 | 2.48 |
| **17** | 0.8043 | 0.1267 | 0.6776 | 0.00 | 0.01 |
| **18** | 0.8051 | 0.1268 | 0.6783 | 0.00 | 0.11 |
| **19** | 0.8143 | 0.1303 | 0.6840 | 0.01 | 0.95 |
| **20** | 0.8063 | 0.1317 | 0.6746 | 0.00 | 0.43 |
| **Average** | 0.81 ± 0.01 | 0.13 ± 0.002 | 0.68 ± 0.01 | 0.009 ± 0.0065 | 1.4 ± 0.96 |

W1 = Weight of filled capsule; W2 = Weight of empty capsule; W3 = Weight of capsule content
